# Decomposing Intolerance of Uncertainty: No Association With Affective Decision Making in a Community Sample

**DOI:** 10.5334/cpsy.140

**Published:** 2025-09-18

**Authors:** Yannik Paul, Anya Pedersen, Kamil Fuławka

**Affiliations:** 1 Christian-Albrechts-University of Kiel, Germany; 2 Max-Planck Institute for Human Development, Berlin, Germany; 3 TUD Dresden University of Technology, Dresden, Germany

**Keywords:** Intolerance of Uncertainty, Decision-making, Information sampling, Description-Experience Gap, Loss Aversion, Probability Weighting

## Abstract

Intolerance of Uncertainty (IU) is a transdiagnostic factor in psychological disorders, yet its underlying psychological mechanisms remain unclear. To close this gap, we first identify three potential mechanisms from existing definitions of IU: (1) negativity overweighting, (2) probability distortion, and (3) information deficit aversion. Second, we demonstrate how these mechanisms map onto well-established preference patterns in decision making under uncertainty as captured by Cumulative Prospect Theory: (1) loss aversion, (2) nonlinear probability weighting, and (3) the description–experience (DE) gap. Third, we conduct an affective decision-making experiment to investigate the relationship between self-reported IU and these preference patterns, as measured with individually estimated parameters of cumulative prospect theory. In the study, 100 participants made 120 choices between hypothetical painkillers with different probabilistic side effects. Half of the choices were made in a description condition, where all information was provided upfront; the other half in an experience condition, where participants acquired information through sampling. Trait IU was measured with a questionnaire. Participants overweighed side effects relative to treatment benefits (loss aversion), overestimated the probability of unlikely negative outcomes (increased nonlinear probability weighting), and their probability weighting patterns differed between the experimental conditions (DE gap). However, their preference patterns did not correlate with IU scores. Possible explanations are that the task did not effectively establish an affective context with real consequences for behavior, or that disorder-specific processes were not captured in our community sample. These findings highlight the need for a precise definition of IU and suggest avenues for designing tasks that enable a better understanding of IU.

## 1 Introduction

To function effectively in a complex world, individuals must constantly make decisions, often under conditions of uncertainty. For example, outcomes may be probabilistic, such as when people invest on the stock market, or relevant information may be missing, such as when people decide between medical treatments without knowing all the potential side effects and their likelihoods (Bradley & Drechsler, 2013; Carleton, 2016a, 2016b; Rowe, 1994).

Individual differences in intolerance of uncertainty (IU) — that is, in how people react cognitively, emotionally, and behaviorally to uncertainty (Freeston et al., 1994) — have been studied extensively in clinical psychology. Originating from research on generalized anxiety disorder (Shihata et al., 2016), IU has emerged as a transdiagnostic risk factor for a range of mental health disorders (Gentes & Ruscio, 2011; Holaway, Heimberg & Coles, 2006; McEvoy & Mahoney, 2013; Vander Haegen, Etienne & Duregger, 2016). It is associated with disorder-specific symptoms, such as intrusive thoughts in obsessive-compulsive disorder and panic attacks in panic disorder (Gentes & Ruscio, 2011; McEvoy & Mahoney, 2012), can be alleviated through standard treatments for these disorders (Wilson, Abbott & Norton, 2023), and is related to other transdiagnostic constructs like repetitive negative thinking or ineffective emotion regulation (McEvoy & Mahoney, 2013; Sahib et al., 2023). It is also observed in subclinical expressions in undergraduate and community samples (Bottesi et al., 2016; Bottesi et al., 2019; Ouellet et al., 2019). With research increasingly emphasizing the need for treatment approaches that specifically target IU (Einstein, 2014; McEvoy & Erceg-Hurn, 2016), it is crucial to understand the psychological mechanisms behind it (McEvoy & Erceg-Hurn, 2016; Starcevic & Berle, 2006).

Most research on IU has focused on associations between self-report measures of IU and other self-report measures. This approach, while valuable for establishing the construct’s clinical relevance, fails to shed light on the psychological mechanisms underlying IU. This would require observing concrete behavior in situations involving uncertainty. Although IU has been linked to associative learning of threat and safety (Morriss, Christakou & van Reekum, 2016; Morriss et al., 2019), this work primarily targets low-level contingency learning and may not capture the more complex decisions often described in IU theory. In these later stages of decision making (Bach & Dolan, 2012), evidence on how IU influences behavior remains inconclusive (Carleton et al., 2016; O’Bryan et al., 2021), with some studies finding reliably associations of behavior and self-reported IU (Ladouceur, Talbot & Dugas, 1997; Jacoby et al., 2014) and others finding only small or no associations (Carleton et al., 2016; Morein-Zamir et al., 2020). Several authors have noted that more specific and nuanced methods are needed to capture behavior under IU and that, in general, a more mechanistic perspective on IU is required (Carleton et al., 2016; Cobos et al., 2024; O’Bryan et al., 2021; Sandhu, Xiao & Lawson, 2023). Key challenges include inconsistency in definitions of IU (Starcevic & Berle, 2006), including the definitions of uncertainty itself, difficulties in operationalizing and predicting specific behavioral responses (Carleton et al., 2016; O’Bryan et al., 2021), and challenges in conceptualizing the role of affect and threat in IU-related behavior (Jacoby et al., 2016; O’Bryan et al., 2021; Ouellet et al., 2019).

In this study, we investigate how IU, as measured by questionnaires, corresponds to behavior under uncertainty, as measured by a decision task. Although uncertainty has been conceptualized in various ways across different fields (Bach & Dolan, 2012; Bland & Schaefer, 2012), we argue that decision tasks are best suited to operationalize situations hypothesized by the IU literature. To this end, we first identify three distinct psychological mechanisms from commonly used definitions of IU. Second, we introduce cumulative prospect theory (CPT), a framework used in decision science to measure and describe choice behavior under uncertainty, and illustrate how the three psychological mechanisms identified can be mapped onto constructs within this framework. Finally, we develop an experimental paradigm that integrates insights from past research to test hypotheses derived from the CPT framework.

### 1.1 Different definitions of IU lead to different predictions about behavior

We propose that the psychological mechanisms implied by the various definitions of IU can be synthesized into three mechanisms: (1) negativity overweighting, (2) probability distortion, and (3) information deficit aversion (see Table 1; for a related overview, see Carleton, 2012). To illustrate, consider the example of planning a mountain hike when the weather forecast predicts a 5% chance of a storm. A definition focusing on negativity overweighting would predict that a person with high IU might find the small prospect of getting cold and wet so off-putting that they decide not go at all. A definition focusing on probability distortion would suggest that a high-IU individual will overweight the probability of a storm and likewise decide to stay safe at home. A definition focusing on information deficit aversion would imply that a high-IU individual would worry about not knowing, for example, the exact timing of the storm. They might either seek out more information or decide not to set off either.

**Table 1:** Common definitions of IU and the implied psychological mechanism.


	PSYCHOLOGICAL MECHANISM	EXAMPLE DEFINITION	RELEVANT CITATIONS

1	Negativity overweighting	“[…] tendency of an individual to consider it unacceptable that a negative event may occur, however small the probability of its occurrence” (Dugas, Gosselin & Ladouceur, 2001)	(Arditte Hall & Arditte, 2024; Bredemeier & Berenbaum, 2008; Deschenes et al., 2010; Dugas etal., 2005; Morriss et al., 2022)

2	Probability distortion	“[…] tendency to overestimate the chance of and be unwilling to accept potential, but unlikely, negative outcomes in uncertain situations […]” (Donthula et al., 2020)	(Deschenes et al., 2010; Dugas, Buhr & Ladouceur, 2004; Gosselin et al., 2008; Luhmann, Ishida & Hajcak, 2011; Milne, Lomax& Freeston, 2019; Reuman et al., 2015)

3	Information deficit aversion	“[…] individual’s dispositional incapacity to endure the aversive response triggered by the perceived absence of salient, key, or sufficient information, and sustained by the associated perception of uncertainty” (Carleton, 2016b)	(Carleton, 2016a; Freeston & Komes, 2023; Grenier, Barrette & Ladouceur, 2005; Ladouceur, Talbot & Dugas, 1997; Milne, Lomax & Freeston, 2019; Pepperdine, Lomax & Freeston, 2018)


**Figure 1:**
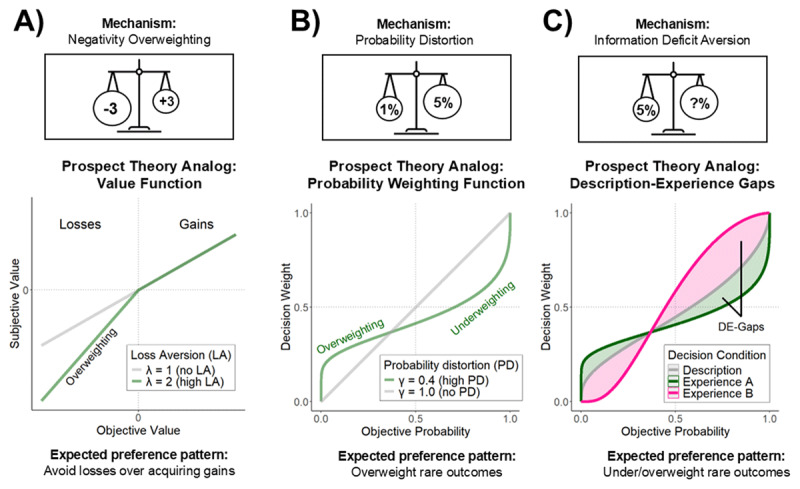
**Cumulative prospect theory and the psychological mechanisms underlying Intolerance of Uncertainty. A)** Negativity overweighting can be captured in CPT by loss aversion, measured with the λ parameter, **B)** probability distortion can be captured in CPT by the shape of the nonlinear probability weighting function, measured with the γ parameter, **C)** information deficit aversion aligns with the Description–Experience gap (DE gap), which can be captured in CPT by the difference in probability weighting between decisions from description and decisions from experience. Depending on task structure and analysis strategy, probability weighting in decisions from experience can over- or underweight probabilities.

These psychological mechanisms correspond to psychological mechanisms assumed in Cumulative Prospect Theory (CPT), one of the most prominent formal models of choice under risk and uncertainty (Tversky & Kahneman, 1992), being widely used in both economic and medical contexts (Schwartz, Goldberg & Hazen, 2008; Verma, Razak & Detsky, 2014). Next, we illustrate how decision science and CPT provide a framework for measuring choice behavior that can be leveraged to investigate how IU shapes decision making under uncertainty. To this end, we discuss the mapping between CPT and the psychological mechanisms underlying IU (see Figure 1 for a graphical representation).

### 1.2 Decision making under risk and uncertainty

Clinical research on IU rarely distinguishes between different forms of uncertainty and risk. Decision science, in contrast, clearly differentiates between certainty, uncertainty, and risk (Mousavi & Gigerenzer, 2014). *Certainty* refers to situations where outcomes occur with 100% probability. Under *risk*, outcomes are known and probabilistic, and their probabilities are known. Under *uncertainty*, outcomes are known and probabilistic, but their probabilities are unknown. Over the decades, decision science has identified a number of regularities in how people make choices under risk and uncertainty, often by investigating choices involving monetary lotteries (see Keller, 1992, for examples).

A monetary lottery consists of a set of probabilistic outcomes, for example, an 80% chance of winning $100 (gains) versus a 20% chance of losing $200 (losses). In a standard decision task, participants make multiple choices between carefully designed pairs of lotteries, typically revealing consistent preference patterns (e.g., overweighting certain outcomes or changing preferences the same problem scaled differently). The observed preference patterns have motivated the development of formal models of risky choice, including CPT (Tversky & Kahneman, 1992). CPT uses nonlinear functions to transform objective outcomes and probabilities into nonlinear subjective representations. The functions were designed to capture the most commonly observed preference patterns and are assumed to reflect distinct psychological mechanisms. The free parameters of these functions, estimated separately for each individual, serve as indirect measures of the assumed underlying psychological mechanisms, therefore summarizing the aforementioned preference patterns in these parameters. In this study, we focus on three well-researched preference patterns: *loss aversion, nonlinear probability weighting* (Tversky & Kahneman, 1992), and the *description–experience gap* (DE gap) (Hertwig et al., 2004; Hertwig & Erev, 2009). We now introduce these preference patterns, demonstrate how they are captured in CPT via parameters and discuss how they relate to the proposed psychological mechanisms of IU (see Figure 1 for an overview).

*Loss aversion* describes the tendency for individuals to be more sensitive to potential losses than to potential gains (Tversky & Kahneman, 1991). In a classic example, Tversky and Kahneman (1992) found that participants required a potential gain of at least $202 at 50% probability to offset a potential loss of $100 at the same probability. In other words, on average, monetary losses seem about twice as impactful as equivalent monetary gains. In CPT, individual levels of loss aversion are represented by the value function, and the λ parameter determines how much losses are overweighed relative to gains. Increased values in λ above one would lead to a preference pattern where losses avoided even though equal gains would be available. Loss aversion corresponds to the first psychological mechanism of IU, negativity overweighting (Table 1), where the negative outcome itself triggers IU-related processes (see Figure 1a).

*Nonlinear probability weighting* describes how people tend to choose between monetary lotteries as if they were overweighting small probabilities and underweighting large probabilities (Kahneman & Tversky, 1979; Tversky & Kahneman, 1992). For example, in Kahneman and Tversky (1979), 83% of participants preferred a 100% chance to lose $5 over a 0.1% chance to lose $5000 despite the statistical equivalence of both options. This example illustrates how small probabilities are overweight relative to their objective value. This phenomenon aligns with the second psychological mechanism of IU, probability distortion, which involves cognitive biases in processing small and large probabilities. Specifically, it refers to the tendency to overweight the small probabilities of unlikely outcomes and to underweight large probabilities (see Figure 1b). Values lower than one in γ would lead to a preference pattern where rare events have more impact on decisions, for example avoiding an impactful but very unlikely loss. In CPT, individual differences in nonlinear probability weighting are represented by the probability weighting function. As γ approaches 1, the probability weighting function becomes more linear, thus probability sensitivity increases. When γ diverges from 1, and approaches 0 or infinity, the function becomes more inverted S-shape or S-shape, respectively.

*The description–experience gap (DE gap)* refers to reversals in preferences between choices made under risk and uncertainty (Hertwig & Erev, 2009). In choices under risk, participants are given all relevant information about a lottery’s outcomes and their probabilities in text form (i.e., *description* condition). In choices under uncertainty, lotteries are presented in the form of interactive “buttons”—participants click on the buttons to sample outcomes from the respective option according to the coded probabilities. By sampling from the lottery many times (i.e., *experiencing* it), participants can discover the available outcomes and estimate their probabilities based on how often they occur. One well-known preference reversal pattern is that, in the description condition, most people prefer $3 for 100% (sure option) to $4 with 80% and $0 otherwise (risky option). However, when given the chance to “experience” both lotteries through simulations, most people prefer the risky option. Initially, it was thought that the DE gap was caused by the underweighting of rare events and recency effects (Hertwig et al., 2004). Underweighting of rare events occurs because people usually take relatively few samples in the experience condition, resulting in biased representation of rare events. Recency effects arise when more recent outcomes have a stronger influence on choice behavior than do earlier ones. However, a recent meta-analysis showed that the DE gap holds even when the experienced probabilities match the described ones, and that recency effects likely reflect strategic sampling termination (Wulff, Mergenthaler-Canseco & Hertwig, 2018). Thus, the exact cause of the DE gap remains an open question.

Nevertheless, the DE gap is inherently linked to choice behavior under incomplete information, which closely aligns with the third potential psychological mechanism—information deficit aversion (see Figure 1c). The DE gap can be measured in various ways (Wulff, Mergenthaler-Canseco & Hertwig, 2018); here, we compare individual probability weighting functions estimated separately for the same choices made twice: Once under a description condition and once under an experience condition. This approach has been successful in other studies involving decision problems with two risky alternatives (e.g., Glöckner et al., 2016; Kellen, Pachur & Hertwig, 2016). It highlights the different ways individuals use probability information in these two modes of decision making (Hau et al., 2008). A simpler way to measure the DE gap is by looking at the proportion of preference reversals, as described in the previous paragraph. Finally, given that IU has been linked to information gathering (Jacoby et al., 2016), it is reasonable to also expect behavior in the experience condition to be linked to information deficit aversion. Thus, both probability weighting in the experience condition and sampling behavior are also metrics of interest.

Despite the parallels between IU and these three well-established preference patterns in decision making, no previous studies have, to our knowledge, empirically examined these relationships. Studying the relations between the preference patterns and IU could provide insights into the validity of different IU definitions, and help determine which psychological mechanisms best represent IU as measured by self-reports. Previous attempts to link choice behavior with IU have produced inconsistent results (Carleton et al., 2016; O’Bryan et al., 2021). In the next section, we analyze these findings to identify potential reasons for the inconsistencies. Our aim is to guide the development of an ecologically and theoretically robust task within the frameworks of behavioral decision science and CPT.

### 1.3 Past attempts at operationalizing IU

Various paradigms have been used to operationalize uncertainty-related behavior (Carleton et al., 2016). One of the most popular tasks to operationalize outcome uncertainty in decision making behavior (Bach & Dolan, 2012) is the beads task (Huq, Garety & Hemsley, 1988), where participants infer the ratio of black and white beads in a container by drawing a series of random beads from it. Participants continue drawing samples until they feel ready to decide. Outcomes are behavioral measures like the number of draws to decision, decision time, and task performance, as well as negative affect and state uncertainty during and after the task (O’Bryan et al., 2021). However, findings on the relationship between decision behavior in the beads task and IU are mixed. Some studies have shown associations with IU or constructs close to IU (Jacoby et al., 2014; Ladouceur, Talbot & Dugas, 1997); other studies have failed to replicate these findings (Jacoby et al., 2016; Morein-Zamir et al., 2020; Osmanağaoğlu et al., 2021). Studies using different paradigms have found small, if any, relationships between concrete decision behavior and IU (Carleton et al., 2016; Cobos et al., 2024; Morriss, Christakou & van Reekum, 2016; O’Bryan et al., 2021). In two seminal studies, risk aversion but not loss aversion was associated with GAD, anxiety and worry (Charpentier et al., 2017; Sporrer et al., 2024), which indicate that the psychological mechanisms captured by CPT might also play a role in IU.

It has been suggested that several factors may affect how IU shapes behavior, namely, perceived threat, negative affect during and after the task, as well as the affective context of the task itself (Carleton et al., 2016; Jacoby et al., 2016; O’Bryan et al., 2021). Supporting this, models of IU suggest that uncertainty, especially when it relates to a potentially aversive event, triggers a negative emotional response (Anderson et al., 2019; Carleton et al., 2012; Einstein, 2014). The degree of this negative affect varies with individual levels of IU. When these negative emotions are not regulated adaptively, more maladaptive coping strategies and reactions emerge (Sahib et al., 2023). These maladaptive coping mechanisms can manifest as approach behaviors (e.g., checking, information gathering, worry) or avoidance behaviors (e.g., decision paralysis, safety behaviors) (Birrell et al., 2011; Sahib et al., 2023). In summary, theoretical considerations and empirical findings suggest that the affective aspects and contexts of IU are important for understanding the mechanisms by which IU affects behavior.

### 1.4 Summary of research goals

In this study, we aim to uncover the psychological mechanisms underlying IU by using a paradigm that captures decisions made under risk and uncertainty. We identify three psychological mechanisms potentially underlying IU: negativity aversion, probability distortion, and information deficit aversion. These categories map closely to preference patterns captured in CPT: loss aversion, nonlinear probability weighting, and the DE gap. A review of theoretical accounts and previous efforts to operationalize IU-related choice behavior emphasizes the critical role of affect and context. The primary objective of this study is to identify which preference patterns, as measured by CPT, correlate best with self-reported IU, enabling us to evaluate which psychological mechanisms best represent the concept of IU. To this end, we employ a correlational design, and a decision task focused on affective outcomes rather than monetary lotteries. Specifically, the task involves medical decisions between hypothetical medications (similar to the approaches taken in Lejarraga et al., 2016; Pachur, Hertwig & Wolkewitz, 2014; Suter, Pachur & Hertwig, 2016). In summary, this study aims to investigate which psychological mechanisms of decision making under uncertainty are associated with IU as assessed through self-report measures.

## 2 Methods

The Study was preregistered on AsPredicted (#176194, aspredicted.org/8bvq-48vh.pdf). The study was conducted in German.

### 2.1 Participants

We recruited 101 participants via the Prolific Platform. Due to a technical error, this exceeded the intended sample size by one. Participants were required to be at least 18 years old, reside in Germany, and speak German fluently, as specified by filters on Prolific. To ensure data quality, participants needed a minimum approval rate of 90%–100% on the platform. One participant was excluded based on preregistered criteria because they did not sample at all in the experience condition, resulting in a final sample of 100 participants (51 female, 49 male; mean age = 31 years, *SD* = 9). The median completion time was 43 minutes. Participants received a remuneration of £9. Informed consent was obtained from all participants after they had read detailed study information, including goals, procedures, data handling, remuneration on Prolific and GDPR rights.

### 2.2 Ethics

The study was approved by the Institutional Review Board (Ethics Committee) of the Max-Planck Institute for Human Development and was conducted in accordance with the Declaration of Helsinki.

### 2.3 Procedure

The study consisted of three main parts. After a brief introduction, participants first completed questionnaire measures. Second, they completed the affective decision task in two conditions (experience and description), with the order of conditions randomized. Third, participants completed the affect rating task, so that complete information about all side effects and pain scenarios would not interfere with the decision tasks. Finally, they were debriefed and redirected to Prolific for remuneration.

### 2.4 Materials

#### 2.4.1 Questionnaire measures

We used the German version of the Intolerance of Uncertainty Scale-18 (IUS-18) to assess trait IU (Buhr & Dugas, 2002; Gerlach, Andor & Patzelt, 2008). The German version consists of 18 items with a factor structure differing from the original IUS, measuring the factors “Impaired ability to act under IU,” “Distress under IU,” and “Vigilance under IU.” A total score can also be calculated. Items were rated on a 5-point Likert scale from “not true” to “completely true”. Emotion regulation, an important covariate of IU, was measured using the German version of the Difficulties in Emotion Regulation Scale (DERS) (Gratz & Roemer, 2004; Gutzweiler & In-Albon, 2018; Ehring et al., 2013), which consists of 36 items rated on a 5-point Likert scale from “almost never” to “almost always”. Both questionnaires were implemented using the survey software LimeSurvey.

#### 2.4.2 Affective decision task

We employed an affective decision-making paradigm implemented in PsychoPy on the Pavlovia Platform (Peirce et al., 2019). The tasks involved choosing between two different painkillers for hypothetical pain scenarios, resembling prior tasks from the literature (Fulawka & Pachur, 2022; Suter, Pachur & Hertwig, 2016). All participants completed this decision-making paradigm in two conditions: A description condition, where all information (treatment success rate; side effects and their likelihood) was provided upfront, and an experience condition, where the information was not provided, but the participant had to infer it via sampling from a distribution mirroring the information displayed in the description condition. Each decision problem was characterized by a hypothetical pain scenario, described by its location and intensity, and two painkiller options. Each painkiller option differed in its treatment success rate (50%–95%), side effect and side effect likelihood (1%–30%). In both conditions, participants were instructed to imagine they suffer from the specified pain and to choose which painkiller they would prefer under such circumstances.

**Figure 2:**
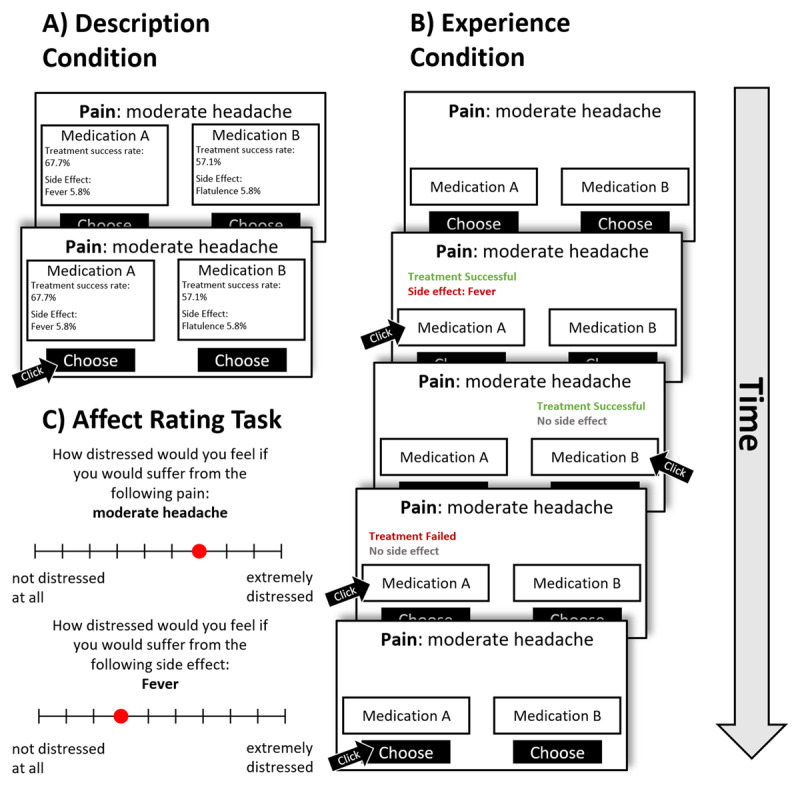
**Affective decision task. A)** Schematic screen in the description condition. Participants choose medication A or B, based on the provided descriptions of consequences, by clicking the respective button. **B)** Schematic screen in the experience condition. Participants click on the boxes to “experience” possible the consequences (i.e. sample from) for each medication. In this example, the participant sampled thrice, twice from medication A (with different outcomes), and once from medication B. Participants could sample as much as they wanted before making the decision. Note that no feedback was provided after choices in both conditions; each participant completed both versions of the task. **C)** After both tasks, participants rated pains and side effects for distress according to a ten-point Likert-scale.

In the description condition (see Figure 2a), all information about painkillers was provided to the participant. Participants were free to consider both options and review the information provided without time constraints. Once they selected either option A or B, no further feedback was displayed and the subsequent trial was presented.

In the experience condition (see Figure 2b), no information about the painkillers was presented initially. Instead, participants could sample outcomes by clicking on visual representations of the painkillers. Each click simulated a single trial of medication use, yielding binary feedback on treatment success and side-effect occurrence. These outcomes were probabilistically determined based on the same parameters used in the description condition but remained undisclosed. Through repeated sampling, participants could estimate the underlying probabilities to inform their choice. Sampling was unconstrained in duration and frequency, allowing participants to gather as much information as they deemed necessary prior to making a decision. After the decision, no further feedback was provided and the subsequent trial began. A computerized tutorial preceded the task to ensure comprehension of the sampling procedure and task structure.

Each condition consisted of 60 choice problems. Pain locations and intensity were informed by common clinical classification categories (Baeyer et al., 2011; Bijur, Latimer & Gallagher, 2003); side effects were taken from Suter, Pachur & Hertwig (2016). In total, 10 unique pain location-intensity pairs and 10 unique side-effects were included in the problem set (for details see Tables SI4-SI6). The intensity ratings of side effects and pains used to construct the problem were obtained by a small pilot study using only the affect rating task, additionally we report rating data from the present study in Figure SI4 and SI5 in the supplement. Order of choice problems and option locations on screen were randomized within conditions. For both tasks, we recorded choices and decision times. In the experience condition, we also recorded each sample and its corresponding timestamp within the trial.

#### 2.4.3 Affect rating task

Participants provided an affect rating for each outcome (i.e., each pain and side effect) presented in the affective decision task. First, they rated each pain on the distress it would cause them on a 10-point Likert scale, ranging from “not at all distressed” to “extremely distressed.” Second, they rated each side effect on the same scale.

### 2.5 Analytical approach

#### 2.5.1 Data processing

Data preprocessing involved several steps to ensure the accuracy and consistency of the analysis. For questionnaires, total questionnaire scores were calculated by summing the individual item scores, after reverse scoring inverted items.

For the experience condition of the affective decision task, we calculated multiple metrics related to sampling behavior. First, the median number of samples was calculated trial-wise for each participant. Second, we quantified participants’ switching behavior between medication A and B during sampling. For each trial, relative switching was computed as the number of observed switches divided by the maximum possible number of switches, given the total number of samples. These trial-level values were then averaged across trials for each participant. All preprocessing and data visualization was done in R (R Core Team, 2022).

#### 2.5.2 Estimation of prospect theory parameters

Participants’ choices in the affective decision task were modelled with Bayesian hierarchical implementation of CPT (Kahneman & Tversky, 1979; Tversky & Kahneman, 1992). In CPT, the subjective value of a choice option is given as the sum of all outcomes multiplied by their respective (transformed) probabilities. In our implementation, the subjective value of a painkiller A for participant *i*, denoted *V*_*i,A*_ was given with:


1 \[
{{V}_{i,A}}\ =\ v\left({{a}_{i,b}} \right)\pi \left({{p}_{b}} \right)+ v\left({{a}_{i,se}} \right)\pi \left({{p}_{se}} \right),
\]

where *a*_*i,b*_ = {1, …, 10} and *a*_*i,se*_ = {–10, …, –1} are the individual’s *i* affect ratings of the pain that is to be treated, and of the painkiller’s side effect, respectively; the *p*_*b*_ and *p*_*se*_ are corresponding probabilities of these events.

The value function *v*(*a*) for the affect ratings has two different cases, depending on the input being side effects *a*_*i,se*_ or benefits *a*_*i,b*_:


2 \[
v\left( a \right) = \left\{ {\begin{array}{*{20}{c}}
{\;a,\;{\mathrm{for}}\;a\; = \;{a_{i,b}}}\\
{ - {\lambda _i}a,\;{\mathrm{for}}\;a\; = \;{a_{i,se}}}
\end{array}} \right.,
\]

Where the *λ*_*i*_ parameter is a captures individual’s *i* loss aversion — the overweighting of negative consequences (here: side effects) relative to gains (here: pains to be treated). With *λ*_*i*_ = 1, losses and gains are weighted equally. For *λ*_*i*_ > 1, side effects are overweighed relative to gains, indicating loss aversion.

The probability weighting function *π*(*p*) transforms objective probability values into decision weights. In the main analysis, we used the formulation developed by Prelec (1998):


3 \[
\pi \left(p \right)={{e}^{-{{\left(-\ln \left(p \right) \right)}^{{{\gamma}_{i}}}}}}
\]

The parameter *γ*_*i*_ captures the degree of an individual’s probability distortion.Values of *γ*_*i*_ < 1 indicate overweighting of small probabilities and underweighting of the large ones, whereas *γ*_*i*_ = 1 indicates objective probability processing (i.e. no probability weighting). In the experience condition, instead of objective probabilities we used the individually experienced proportions of events (e.g., if sampling resulted in observing side effects occurrence in 2 out of 10 instances, the ratio 2/10 was used in modelling as event probability). Note that multiple forms for the weighting function were proposed (see Stott, 2006). In the supplement we show that the four most popular probability weighting functions (including Prelec’s) outperform the linear model (see Figure SI1) and that our main results do not depend on the specific choice of the weighting function (see Table SI3).

The probability of an individual *i* choosing the painkiller *A* over painkiller *B*, denoted *P*(*A*|{*A, B*}), was given by:


4 \[
P\left(A|\left\{ A,\ B \right\}\right)=\frac{1}{1+\exp \left(-{{\theta}_{i}}\left({{V}_{i,A}}-\ {{V}_{i,B}}\right)\right)},
\]

The parameter θ_*i*_ denotes a choice sensitivity parameter and captures to what extend the differences in subjective valuations of the painkillers map onto the choice probability. The parameter thus controls to what extent individual’s *i* choices align with CPT and is therefore strongly associated with model fit and psychological factors related to model fit. Within CPT, theta does not have a specific mechanistic role; we therefore report results concerning theta only in the supplement.

For each participant, the loss aversion *λ*_*i*_, probability weighting *γ*_*i*_, and choice sensitivity θ_*i*_ parameters were estimated separately for description (*λ*_*i,D*_, *γ*_*i,D*_, *θ*_*i,D*_) and experience conditions (*E, γ*_*i,E*_, *θ*_*i,E*_). The individual-level DE-gap was thus measured with the difference *γ*_*i,D*_ – *γ*_*i,E*_ in probability weighting between conditions.

In hierarchical models, the individual parameters are assumed to come from population-level distributions, with parameters of these distributions also estimated from the data. Here we assumed that each pair of individual parameters (e.g., *γ*_*i,D*_ and *γ*_*i,E*_) comes from bivariate normal distributions. To efficiently implement the model in STAN language (Stan Development Team, 2024b), we used the non-centered parametrization and probit transformation of the individual parameters to assure that they stay in desired range. Specifically, using the individual parameter *γ*_*i,D*_ as an example, it was given with:


5 \[
{{\gamma}_{i,D}}=\phi \left(\mu _{\gamma,D}^{\Phi}+{{\xi}_{\gamma i,D}} \right)\times M,
\]

Where the ϕ() function is the standard normal CDF, which maps real values into the [0,1] range and *M* is a scaling factor that sets the upper boundary of the individual parameter. In all analyses and for all parameters we set *M* = 5, allowing for relatively high levels of loss aversion for the *λ* parameters, and for the entire range of shapes for the probability weighting function governed by the *γ* parameter. The population-level mean μ*_γ_*_*,D*_) of the individual parameters *γ*_*i,D*_ is obtained by applying the probit transformation and scaling factor to the mean component within the ϕ() function: \[
{{\mu}_{\gamma,D}}={{\Phi}^{-1}}\left(\mu _{\gamma,D}^{\Phi}\right)\times M
\] — these are the values that we report in the results section.

The term \[
{{\xi}_{\gamma i,D}}
\] is the individual *i*’s displacement from the population-level mean. We assumed that these displacements come from bivariate normal distributions for corresponding pairs of parameters between the experimental conditions:


6 \[
\left(\begin{array}{c} {{\xi}_{\gamma i,D}} \\ {{\xi}_{\gamma i,E}} \\\end{array} \right)\ \sim\ N\ \left(\left[\begin{array}{c} 0 \\ 0 \\\end{array} \right],\ \left[\begin{array}{c} \sigma _{\gamma,D}^{2} & {{\sigma}_{\gamma,DE}} \\ {{\sigma}_{\gamma,DE}} & \sigma _{\gamma,E}^{2} \\\end{array} \right] \right),
\]

where \[
\sigma _{D}^{2}
\] and \[
\sigma _{E}^{2}
\] are the variances of individual-level displacement distributions in the description and experience conditions, respectively, and σ*_γ_*_*,DE*_ is the covariance. The multivariate binding of corresponding individual parameters allows for estimation of individual parameters in both conditions, while preserving the within-individual dependence of the parameters. In STAN, the estimation of the variance covariance matrix was done using optimization through Cholesky factorization (Stan Development Team, 2024b).

The model implementation has several advantages. First, it results in efficient and well converging procedure for estimating the posterior distributions of both individual and population-level parameters. Second, for all individual displacements and population mean parameters before probit transformation, we could use standard normal distributions, which after transformation with the ϕ() function resulted in uniform priors for the individual level parameters, not influencing the final shape of the prior distributions. For the correlation coefficient (in the variance-covariance matrix) we used weakly-informative Lewandowski-Kurowicka-Joe (LKJ) prior with parameter η = 4, which assumes that correlations of displacements between conditions to be most likely between –0.5 and 0.5. Thus, overall, the priors in the model were non- or weakly-informative and the estimation was most sensitive to the actual data.

To summarize, for the probability weighing parameter *γ*, the estimated parameters of interest were: the set of individual-level parameters for description and experience conditions, *γ*_*i,D*_ and *γ*_*i,E*_ respectively, the corresponding population-level means μ*_γ_*_*,D*_ and μ*_γ_*_*,E*_, and the correlation between the individual-level parameters (estimated within the variance-covariance matrix) *ρ_γ_*_*,DE*_. The implementation for the estimation of the two other model parameters, namely loss aversion *λ* and choice sensitivity θ was analogous. Note that the Bayesian approach results in estimating posterior distributions of all model parameters, of which we report medians with 95% Bayesian credible intervals (95% BCI; the 2.5% and 97.5% quantiles).

**Figure 3:**
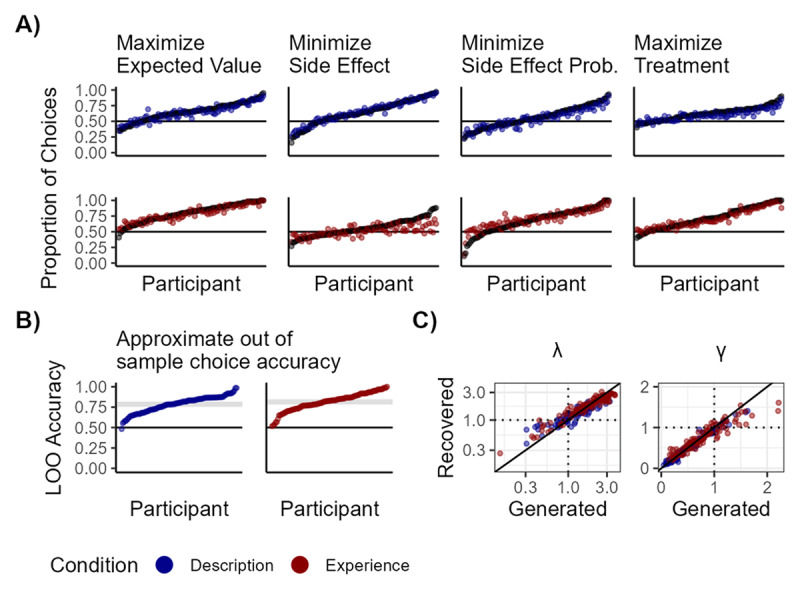
**Summary of model fit. A)** Posterior predictive checks for the CPT model. We defined several decision strategies participants may have used. For each strategy, we coded whether a participant’s choice aligned with the strategy, excluding trials where no optimal option could be determined. The proportion of observed choices consistent with each strategy is shown in black, and the model-predicted probability of choosing the rule-consistent option is shown in blue (description) or red (experience). Maximize expected value = Defined as deciding for the option with the highest sum of probability-weighted benefit and side effect; Minimize Side Effect = Defined as taking the option with the least intense side effect; Minimize Side Effect Prob. = Defined as taking the option with the least probable side effect; Maximize Treatment = Defined as taking the option with the best treatment probability. **B)** Model performance, quantified via LOO-based predictive accuracy. Using leave-one-out cross-validation log-likelihoods, we computed predicted choice probabilities for each trial, converted these to binary predictions using a 0.5 threshold, and calculated accuracy as the proportion of correctly predicted choices per participant. Horizontal lines show mean accuracy for conditions. **C)** Parameter recovery of the model. Correlations between simulated and recovered parameters for description were *r* = 0.92 and *r* = 0.98 for λ and γ respectively. For experience, correlations were *r* = 0.93 and *r* = 0.93 for λ and γ. For details, refer to section 1.2 in the supplement.

The model was written in STAN programming language for statistical computing (Stan Development Team, 2024b) and estimated form R using the rstan-package (Stan Development Team, 2024a). Posterior distributions of parameters were estimated using four chains, each with 1,000 warm-up iterations and 4,000 subsequent samples, totaling 5,000 samples after discarding every other sample. The diagnostic plots and convergence statistics clearly (all R-hats < 1.01) indicated that the sampling procedure resulted in well-mixed chains. Parameter recovery was very good (see Figure 3c). Additionally, all posterior distributions were unimodal.

#### 2.5.3 Statistical analyses

Bayesian nonparametric tests of Spearman’s *ρ* were used for correlation analysis (van Doorn et al., 2020), with uniform uninformative beta priors. These non-parametric tests output a Bayes Factor (BF_10_) as a measure of evidence for the alternative hypothesis (BF_10_ > 1), or the null hypothesis (BF_10_ < 1). Medians from individual parameter posteriors were used for correlational analysis and plots.

## 3 Results

We first report descriptive statistics, demonstrating that the sample displayed meaningful choice patterns in both conditions of the task. Next, we present results from a posterior-predictive check that demonstrates the CPT model accurately captures these meaningful choice patterns as well as model fit and parameter recovery. After that, we describe sample characteristics with respect to loss aversion, nonlinear probability weighting, and the DE gap. Subsequently, we present results addressing our research question: Which psychological mechanisms of decision making under uncertainty are associated to IU as measured with self-reports?

### 3.1 Descriptive analysis

As expected, IUS scores were highly correlated with DERS scores (*ρ* = 0.58, BF_10_ > 1000). IUS scores were concentrated in the lower half of the scale (*M* = 50.55, *SD* = 13.89, see Table 2). Correlations between IUS subscales ranged from 0.55 to 0.69. Correlations of IUS subscales with the DERS were between 0.33 and 0.65. Participants sampled on average 14.5 times, which is less often than in other studies using risky choice problems (Wulff, Mergenthaler-Canseco & Hertwig, 2018), but close to similar tasks in the medical decision domain (Lejarraga et al., 2016). Mean switching was low (*M* = 0.25, *SD* = 0.28), indicating that participants mostly sampled from one option before switching to the other and subsequently making a decision. In the description and experience conditions, participants chose the medication with the highest (described/experienced) weighted average of treatment probability and side effects in 65% and 75% of choices, respectively, and the option with the highest treatment success rate in 62% and 63% of choices, respectively. On the other hand, participants chose the option with the lowest weighted side effect in 61% of choices in the description condition, but in only 48% of choices in the experience condition. To check if the CPT model captures these behavioral patterns, we next analyze if the individual CPT parameters succeed in predicting these choice patterns.

**Table 2:** Descriptive statistics of the Intolerance of Uncertainty Scale (IUS), its subscales and the Difficulties in Emotion Regulation Scale (DERS).


	M	SD	Min	25%	50% MEDIAN	75%	MAX

IUS Total Score	50.55	13.89	22	42.75	50.00	61.25	83

IUS Impaired Ability	14.95	5.38	6	12.00	14.00	19.00	27

IUS Distress under IU	17.50	5.44	8	13.75	17.50	21.00	30

IUS Vigilance	18.10	5.11	6	15.00	18.00	21.00	29

DERS Total Score	93.34	23.53	42	77.75	94.00	113.00	142


### 3.2 Model fit

Model fit is summarized in Figure 3. Overall, the model performed well in capturing participants’ choices. The approximate out-of-sample predictive accuracy of the model was 78% in the description condition and 81% in the experience condition (see Figure 3b). Our sample displayed meaningful choice patterns and use of strategies within both conditions of the experiment, for example preferring positive outcomes and minimizing probabilities of side effects. The model adequately captured these patterns (see Figure 3a). These strategies were not associated with the IUS (see supplement SI2).

### 3.3 Modeling results

**Figure 4:**
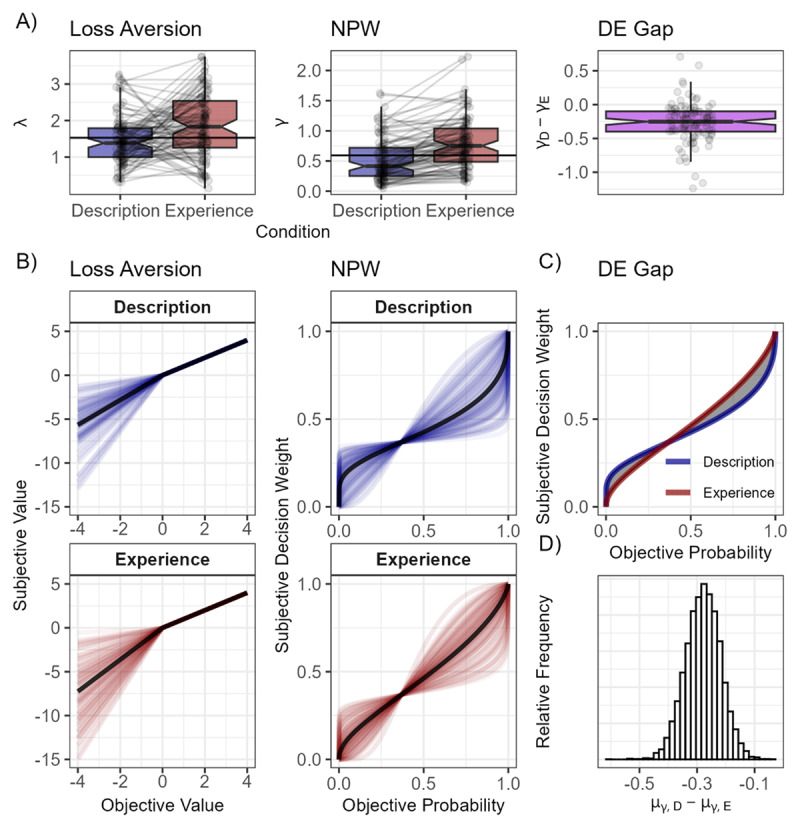
**Overview of modeling parameters. A)** Comparison of individual estimates of loss aversion and nonlinear probability weighting (NPW) between the description and experience conditions, and the individual description–experience gap (DE gap). **B)** Individual estimates of the value function and the probability weighting functions. The black curves represent the function of the median of the posterior estimates of the population-level parameters. **C)** Population-level probability weighting functions (i.e., medians of μ*_γ_*_*,e*_ and μ*_γ_*_*,d*_ estimates). **D)** Population-level posterior distribution of the DE gap. The individual estimates in A and B are the medians of the individual-level posterior distributions; the black lines show medians of corresponding population-level distributions.

An overview of the modeling results is presented in Figure 4. As Figure 4a shows, participants displayed loss aversion and nonlinear probability weighting in both conditions. Loss aversion was higher in the experience condition, μ*_λ_*_*,e*_ = 1.81,95% *BCI*: [1.52,2.13], than in the description condition, μ*_λ_*_*,d*_ = 1.41,95% *BCI*: [1.17,1.68], even though the BCI for the population-level difference distribution of λ in description and experience did marginally include 0, μ*_λ_*_*,d –*_ *_λ_*_*,e*_ = –0.39,95% *BCI*: [–0.79,0.00]. Participants displayed a more linear probability weighting curve (e.g. decreased nonlinear probability weighting) in the experience condition, μ*_γ_*_*,e*_ = 0.71,95% *BCI*: [0.59,0.84], compared to the description condition, μ*_γ_*_*,d*_ = 0.43,95% *BCI*: [0.35,0.52] as indicated by γ values closer to one. This indicates that participants displayed increased probability sensitivity in the experience condition. In line with this, we found a negative DE gap, μ_GAP_ = –0.28,95% *BCI*: [–0.40,–0.17] (see Figure 4c). As seen in Figure 4b, despite these expected patterns on the population level, there was reasonable variance in parameters on the individual level. For example, most of the sample displayed considerable loss aversion, although a small portion of participants also displayed the opposite trend, e.g. underweighting losses. Most of the probability weighting functions of participants were shaped in the inverted s-shape, however a small portion of participants displayed s-shaped probability weighting as well. More s-shaped probability weighting functions are present in the experience condition compared to the description condition. There was very strong evidence for a negative association of the median number of samples and the DE gap (*ρ* = –0.38, BF_10_ = 53.82). For an overview of parameter intercorrelation and choice strategies reported in Figure 3, we refer to the supplement (Figure SI2).

### 3.4 Relationship between IU and decision parameters

**Figure 5:**
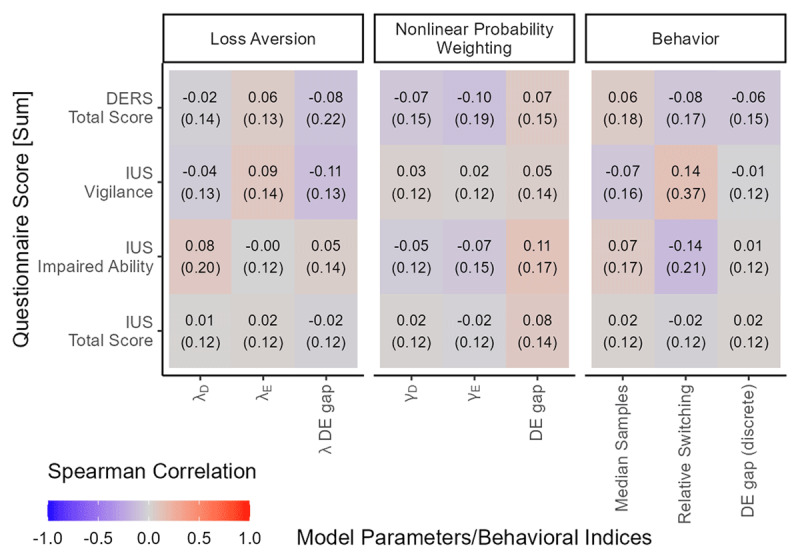
**Correlation Matrix of CPT Parameters and behavioral variables with the intolerance of uncertainty scale (IUS), its subscales and the DERS**. Numbers represent the Spearman correlation, numbers in brackets BF_10_ for undirected Bayesian tests of these correlations. The λ gap is plotted for completeness. The discrete DE gap uses the discrete operationalization (i.e., proportion of preference reversals) from Wulff, Mergenthaler-Canseco & Hertwig (2018).

Across all parameters in all conditions, we found moderate evidence for the null hypothesis that there was no correlation of the IUS score with parameters estimated from choice data (see Figure 5). Loss aversion (λ) was not associated with the IUS score in the description (*ρ* = 0.01, BF_10_ = 0.12) or the experience condition (*ρ* = 0.02, BF_10_ = 0.12). Nonlinear probability weighting (γ) was not associated with the IUS score in the description (*ρ* = 0.02, BF_10_ = 0.12) or the experience condition (*ρ* = –0.02, BF_10_ = 0.13). Neither the DE gap (*ρ* = 0.08, BF_10_ = 0.14) nor median sampling (*ρ* = 0.02, BF_10_ = 0.12) was associated with the IUS score. We additionally tested the discrete operationalization of the DE gap often used in the literature: the proportion of preference reversals (e.g., Wulff, Mergenthaler-Canseco & Hertwig, 2018). Here, a DE gap was identified when participants chose the painkiller with a less probable but worse side effect in the experience condition but rejected it in the description condition. Using this discrete operationalization did not change the results: There was still no association with the IUS score (*ρ* = 0.02, BF_10_ = 0.12). Additional analyses showed that there was no association with IUS subscales (see Figure 5).

## 4 Discussion

Our study aimed to explore the relationship between IU and choice behavior under risk and uncertainty. To this end, we identified three mechanisms potentially underlying IU from commonly used definitions. We then showed how these categories align with preference patterns studied in behavioral decision science and can be operationalized using CPT parameters. Recognizing the importance of affect and threat, we developed an affective decision task with two versions: one measuring decisions under risk; the other decisions under uncertainty. Using CPT, we measured loss aversion, nonlinear probability weighting, and the DE gap, each corresponding to one of the hypothesized psychological mechanisms of IU.

We observed no relationships between self-reported IU and choice behavior, even though participants exhibited strong loss aversion and nonlinear probability weighting in both conditions, and there was evidence of a DE gap.

Our results reveal differences in participants’ decision-making behavior between the description and experience conditions, corresponding to decisions under risk versus decisions under uncertainty within the framework of decision science.

Findings from our affective decision task are consistent with previous results from the medical decision domain. Specifically, Lejarraga et al. (2016) reported lower nonlinear probability weighting in the experience condition than in the description condition of their medical task, while the probability weighting function retained its classic inverted S-shape. Although the level of nonlinear probability weighting was higher in their study than in ours, their task involved fewer decision problems, excluded gains, and included almost no rare outcomes. Our results differ, however, from studies on monetary choices between risky alternatives, which have reported either increased nonlinear probability weighting under experience (Glöckner et al., 2016; Kellen, Pachur & Hertwig, 2016) or an S-shaped probability weighting function (Ungemach, Chater & Stewart, 2009). However, similar to other monetary decision studies, we found more expected value maximizations in the experience condition than in the description condition.

Consistent with Lejarraga et al. (2016), we observed a robust DE gap for medical decisions, even when gains and rare outcomes were included in the choice problems and although both options were risky (Wulff, Mergenthaler-Canseco & Hertwig, 2018). This suggests that affective decisions, as implemented in our task, differ qualitatively from monetary lottery choices. Affective outcomes appear to heighten the influence of uncertainty, resulting in more consistent preferences that are less affected by how choice problems are structured within decision tasks. Future research on choice behavior under IU should therefore carefully design tasks to ensure ecological validity and theoretical relevance by incorporating affective stimuli (e.g., Jacoby et al., 2016).

Although the study provided strong evidence that decision making is influenced by whether participants are dealing with risk or uncertainty, it did find moderate evidence against an association between choice behavior and questionnaire measures of IU. The absence of a clear link between IU and decision-making behavior could be due to several factors that warrant further exploration.

Correlations between behavioral tasks and self-report measures are often weak, both generally (Dang, King, & Inzlicht, 2020) and specifically in the context of risk-taking (Mamerow, Frey, & Mata, 2016). One explanation is that these methods capture different constructs and differ in reliability (Dang et al., 2020). Self-report tools like the IUS-18 assess stable traits, while behavioral tasks, such as the decision-making task used in this study, reflect momentary states and are more sensitive to situational influences. Although this methodological divergence limits the interpretation of our null results, prior research has linked IU to psychopathology (Gentes & Ruscio, 2011) and shown that decision-making tasks can capture core aspects of it (Charpentier et al., 2017; Sporrer et al., 2024). Therefore, limitations of both self-report measures and the behavioral task must be carefully considered when interpreting the present data.

Because the associations of IU and decision making represent trait-state associations, full usage of the spectrum of the self-report measures is needed to enable the detection of these associations beyond the differing scopes of constructs. Therefore, the limited range of IU scores in the present study may have contributed to the null findings. For the range of questionnaire scores, the composition of the sample is crucial: for the present study, participants were recruited through Prolific without clinical preselection. The sample should thus be regarded as a healthy community sample rather than a clinical one that displays IU values in the lower half of the scale. While IU has been shown to influence subclinical symptoms in the general population and undergraduate samples, its effects are most pronounced in patient samples with elevated IU levels with some research even suggesting distinct processes in healthy and patient samples or disorder-specific processes (Shihata, McEvoy & Mullan, 2017; Bottesi et al., 2019). Taken together, these points may have limited the study’s potential to detect meaningful associations or disorder-specific mechanisms and caused subtle but important variations in IU mechanisms to go undetected in our sample. Future research should employ tasks similar to the one used here to operationalize decisions under uncertainty in patient samples. Future versions of the task could also integrate disorder-specific facets of IU, tailoring the scenarios used in the task to disorder-specific topics that could yield additional insights into transdiagnostic and disorder-specific aspects of IU.

Another potential explanation for the absence of a relationship between IU and choice behavior is the limited ecological validity of our task, which may not have succeeded in creating an affective context with real consequences for behavior (Bredemeier & Berenbaum, 2008; Pepperdine, Lomax & Freeston, 2018; Reuman et al., 2015): The pain scenarios in our study were hypothetical and not actually experienced by participants. Future studies could focus on designing tasks with higher ecological validity, incorporating unpleasant stimuli, such as odors or mild electric shocks (von Helversen, Coppin & Scheibehenne, 2020; Stancak et al., 2015). These paradigms could be adapted to introduce tangible consequences to decision-making tasks like ours. Additionally, social situations, which are inherently uncertain, could be employed to establish environments that incorporate both uncertain outcomes and affective consequences (Vives & FeldmanHall, 2018). Additionally, experimental approaches could be employed to overcome the limitations of correlational designs. For instance, state-IU inductions can be used to create high- and low-IU groups in behavioral tasks (Britton & Davey, 2014; Mosca, Lauriola & Carleton, 2016).

We did not include direct comprehension checks in this study. Although such checks could have increased confidence in participants’ task performance and self-reports, our analysis of choice strategies and the posterior-predictive check indicate that most participants employed non-random choice strategies, suggesting they understood the task. Moreover, participants recruited through the Prolific platform are generally known to provide high-quality data (Douglas, Ewell & Brauer, 2023).

Our study found moderate evidence of no association between IU and behavior, though changing the context from risk (where probabilities are known) to uncertainty (where they are not) did influence decision making. Future research should prioritize precise, theoretically informed definitions of IU and uncertainty, and use patient samples and experimental inductions to better capture the nuanced effects of IU on behavior.

## Data Accessibility Statement

The codebase including the modeling Stan code and the data used for the analysis can be found here: https://github.com/y-paul/Decomposing_IU.

## Additional File

The additional file for this article can be found as follows:

10.5334/cpsy.140.s1 Supplementary Information. Supplemental Text, Supplemental Methods and Supplemental Tables.
