# Supplementary material for: Decomposing Intolerance of Uncertainty: No Association With Affective Decision Making in a Community Sample
Source: Comput Psychiatr. 2025 Sep 18;9(1):210–30. doi: 10.5334/cpsy.140 (PMC12447796; doi:10.5334/cpsy.140)
Supplement: Supplementary Information. — Supplemental Text, Supplemental Methods and Supplemental Tables. [file cpsy-9-1-140-s1.pdf]

Supplement: Decomposing Intolerance of Uncertainty: No Association With Affective Decision Making in a Community Sample

Paul, Yannik[1], Pedersen, Anya[1] & Fulawka, Kamil[2,3]

1 Christian-Albrechts-University of Kiel, Germany

2 Max-Planck Institute for Human Development, Berlin, Germany

3 TUD Dresden University of Technology, Dresden, Germany

1 Computational Modeling with Cumulative Prospect Theory

1.1 Model Evaluation

For the posterior predictive checks, we compared model predictions of choice strategies potentially used by participants. These represented heuristics for each choice, for example choosing the option with the least probable side effect. For each participant and trial, we determined whether the actual choice aligned with the choice predicted by a given strategy, excluding trials where the strategy did not identify a superior option. This yielded, for each participant, the proportion of choices consistent with that strategy. Using the model's estimated parameters, we then computed the probability of choosing painkiller A or B, and derived the corresponding probability of following the choice strategy. Figure 3A shows both the observed proportion of strategy-consistent choices and the model-predicted probabilities. A close match between these values indicates that the model successfully captures participants' decision behavior.

For parameter recovery (see Fig. 3C in the main manuscript), we used median estimates of individual parameters estimated from the original data to simulate decisions in both conditions, so that the resulting simulated dataset included exactly the same amount of choices and participants as the original data. We then fitted the model to the simulated data, comparing parameters estimated with the parameters used to simulate the datasets. This procedure was repeated 20 times, and a the median of the medians of the recovered individual posteriors is shown on the y-axis in Figure 3C.

2.1 Alternative Model Versions

The model presented in the main manuscript will be referred to as model A for the following section. To investigate if our results depend on specific form of the probability weighting function, we also estimated CPT with three other commonly used formulations of the probability weighting function. These models were also tested with a control model with no assumed probability weighting (linear).

2.1.1 Model B

For model B, we used a different functional form similar to Gonzalez and Wu (1999) for the probability weighting function :

$$\pi(p) = \frac{p^{\gamma_i}}{p^{\gamma_i} + (1-p)^{\gamma_i}}$$

Where $\gamma_i \in (0,1]$ governs, similar to model A, the degree of probability distortion, with $\gamma_i = 1$ indicating no probability distortion and increased distortion with $\gamma_i < 1$. The value function assumes the same form as in model A.

2.1.2 Model C

For model C, a functional form for the probability weighting function similar to the original version of Tversky and Kahneman (1992) was assumed:

$$\pi(p) = \frac{p^{\gamma_i}}{(p^{\gamma_i} + (1-p)^{\gamma_i})^{\gamma_i^{-1}}}$$

Where $\gamma_i \in (0,1]$ governs, similar to model A, the degree of probability distortion, with $\gamma_i = 1$ indicating no probability distortion and increased distortion with $\gamma_i < 1$. The value function assumes the same form as in model A.

2.1.3 Model D

For model F, a simple power function was assumed for the probability weighting function (Stott, 2006), implying an exponential function instead of an inverted S-shape:

$$\pi(p) = p^{\gamma_i}$$

With the value function assuming the same functional form as model A.

To compare model performance we estimated approximate leave-one-out cross-validation accuracy (LOO accuracy) for each individual participant (Fig. 3B in the main manuscript). LOO accuracy provides an estimate of how well each model predicts unseen data for individual participants. This participant-level evaluation helps assess the generalizability and robustness of each model.

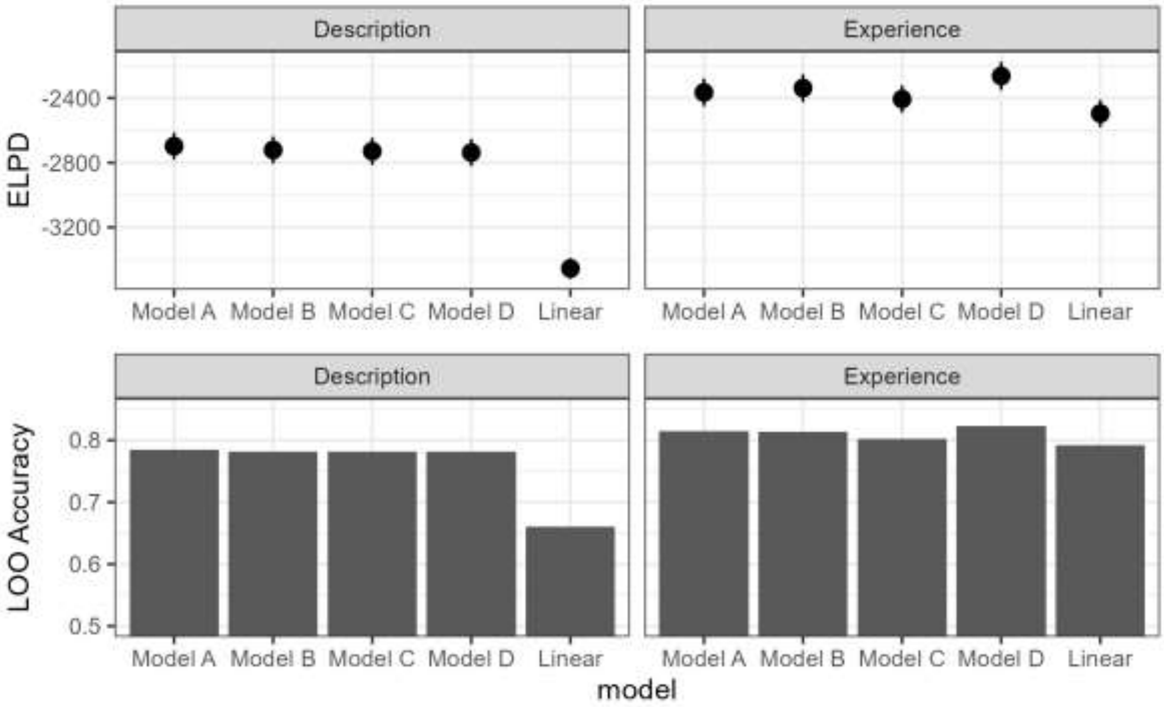

Figure SI1. Model comparison across all estimated models, using expected log predictive density (ELPD) and leave-one-out cross-validation accuracy (LOO accuracy) as primary metrics for evaluating model performance.

2 Supplemental Correlation and Descriptive Results

The theta parameter in Prospect Theory is a scaling factor and therefore strongly associated with model performance (LOO accuracy), $r = .61$ for description and $r = .76$ for experience (model A). Theta is strongly associated with model fit (and with psychological factors related to model fit) but serves no direct mechanistic role within CPT. We therefore report results concerning theta in the following sections.

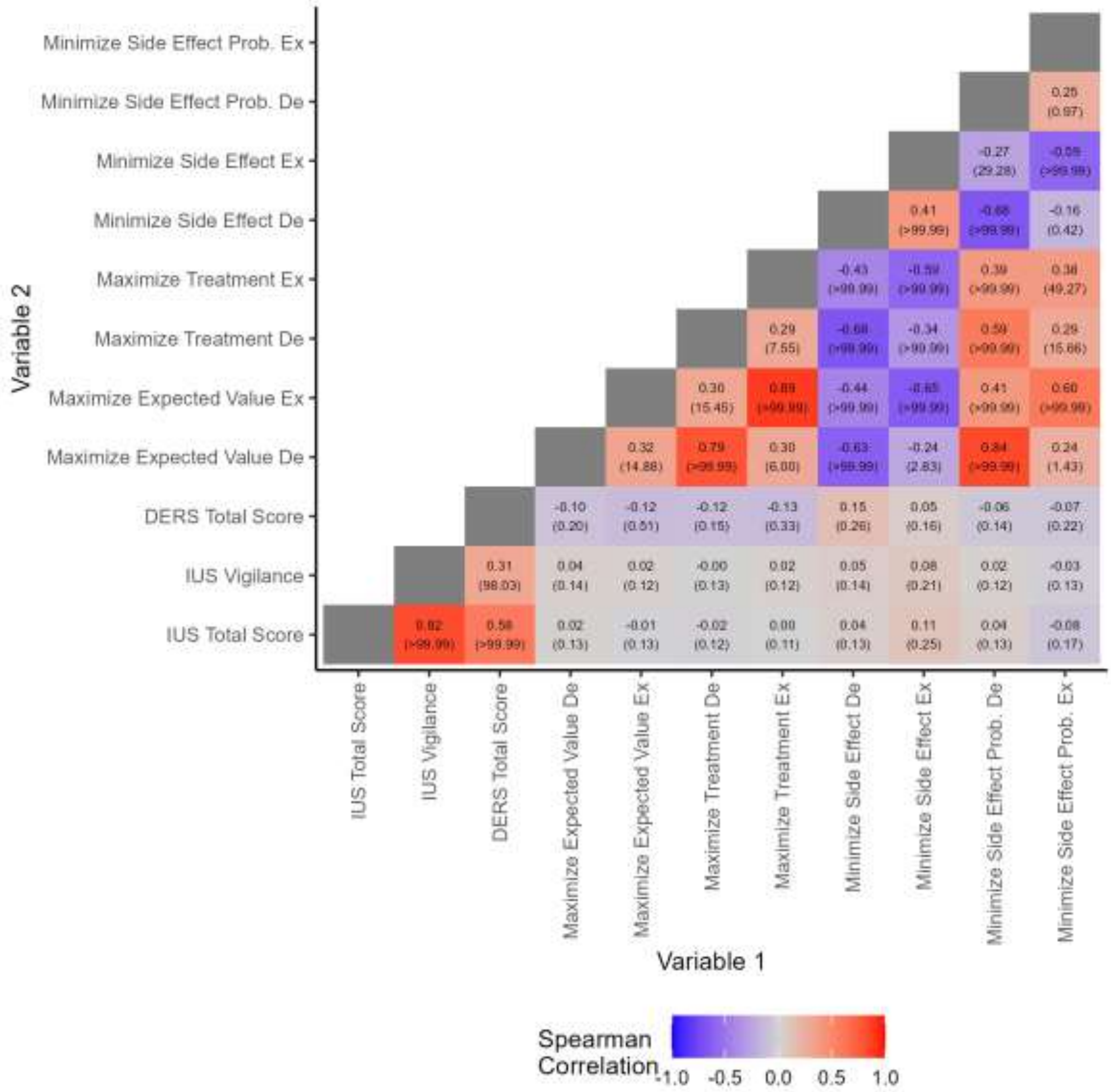

Figure SI2. Correlation heat map of the intolerance of uncertainty scale 18 (IUS), its subscales, and the difficulties in emotion regulation questionnaire (DERS) with choice strategies. Labels within the tiles represent Spearman correlations with the respective BF_{10} in brackets. Maximize expected value = Defined as deciding for the option with the highest sum of probability-weighted benefit and side effect; Minimize Side Effect = Defined as taking the option with the least intense side effect; Minimize Side Effect Prob. = Defined as taking the option with the least probable side effect; Maximize Treatment = Defined as taking the option with the best treatment probability.

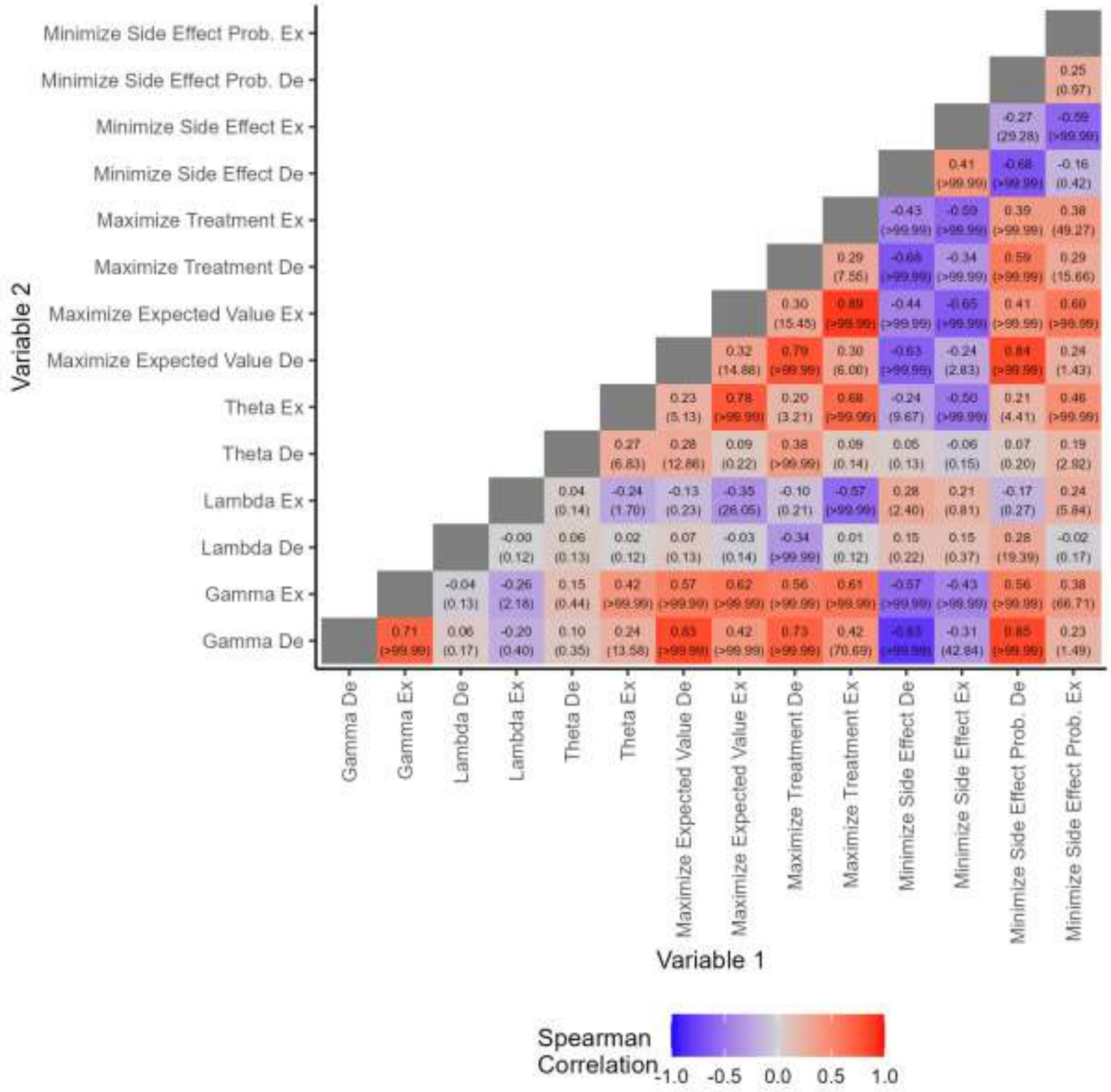

Figure SI3. Correlation heat map of the choice strategies with parameters. Labels within the tiles represent Spearman correlations with the respective BF_{10} in brackets. Maximize expected value = Defined as deciding for the option with the highest sum of probability-weighted benefit and side effect; Minimize Side Effect = Defined as taking the option with the least intense side effect; Minimize Side Effect Prob. = Defined as taking the option with the least probable side effect; Maximize Treatment = Defined as taking the option with the best treatment probability

3 Alternative Model Results

Table SI1.

Medians and 95% Bayesian confidence intervals (95% BCI; the 2.5% and 97.5% quantiles) for the population-level posteriors of parameters lambda (λ) and gamma (γ), the description-experience gap (γ_D - γ_E) and the correlations between individual parameters between conditions of the decision task (ρ_λ, ρ_γ). All parameters are reported for the description (D) and experience (E) condition of the decision task.

Model	λ_D		γ_D		λ_E		γ_E		γ_D - γ_E		$\rho_{\lambda;DE}$		$\rho_{\gamma;DE}$	
	Median	BCI	Median	BCI	Median	BCI	Median	BCI	Median	BCI	Median	BCI	Median	BCI
A	1.41	[1.17, 1.68]	0.43	[0.35, 0.52]	1.81	[1.52, 2.13]	0.71	[0.59, 0.84]	-0.28	[-0.40, -0.17]	-0.17	[-0.28, 0.27]	0.57	[0.33, 0.75]
B	0.89	[0.72, 1.10]	0.35	[0.29, 0.43]	1.64	[1.39, 1.94]	0.66	[0.54, 0.80]	-0.30	[-0.44, -0.19]	-0.01	[-0.28, 0.28]	0.54	[0.30, 0.72]
C	1.85	[1.56, 2.20]	0.50	[0.44, 0.57]	1.97	[1.63, 2.34]	0.83	[0.75, 0.93]	-0.32	[-0.42, -0.24]	-0.25	[-0.25, 0.30]	0.56	[0.28, 0.77]
D	0.43	[0.33, 0.35]	0.28	[0.22, 0.34]	0.76	[0.61, 0.95]	0.40	[0.31, 0.49]	-0.12	[-0.22, -0.03]	-0.03	[-0.31, 0.32]	0.34	[0.07, 0.57]

Table SI2.

Medians and 95% Bayesian confidence intervals (95% BCI; the 2.5% and 97.5% quantiles) for the population-level posteriors of parameter theta (θ) and the correlations between theta between conditions of the decision task (ρ_θ). Theta is reported for the description (D) and experience (E) condition of the decision task.

Model	θ_D		θ_E		$\rho_{\theta;DE}$	
	Median	BCI	Median	BCI	Median	BCI
A	1.87	[1.62, 2.16]	0.93	[0.78, 1.13]	0.17	[-0.13, 0.43]
B	2.29	[1.95, 2.68]	0.96	[0.79, 1.15]	0.11	[-0.22, 0.41]
C	2.12	[1.83, 2.45]	0.88	[0.72, 1.07]	-0.04	[-0.32, 0.24]
D	2.80	[2.38, 3.29]	1.33	[1.09, 1.61]	0.08	[-0.30, 0.44]

Table SI3.

Non-parametric correlations (van Doorn et al., 2020) of medians of individual estimates of parameters lambda (λ) and gamma (γ) and the description-experience gap (γ_D - γ_E) with IU as measured by the Intolerance-Uncertainty-Scale 18 (Gerlach et al., 2008).

Model	λ_D		γ_D		λ_E		γ_E		$\gamma_D - \gamma_E$	
	ρ	BF_{10}	ρ	BF_{10}	ρ	BF_{10}	ρ	BF_{10}	ρ	BF_{10}
A	0.01	0.12	0.02	0.12	0.02	0.12	-0.02	0.12	0.08	0.13
B	0.04	0.13	0.02	0.12	0.02	0.12	-0.03	0.13	0.08	0.16
C	0.05	0.14	0.02	0.12	0.03	0.11	0.13	-0.02	0.13	0.22
D	0.06	0.16	0.03	0.12	0.03	0.12	-0.09	0.16	0.17	0.34

4 Pains and side effects

Table SI4.

The following tables presents all pains and side effects used in the decision task in both versions.

Pains

ID	German	English
1	leichte Halsschmerzen	mild sore throat
2	leichte Rückenschmerzen	mild back pain
3	leichte Kopfschmerzen	mild head ache
4	mittlere Schmerzen im Brustkorb	moderate chest pain
5	mittlere Halsschmerzen	moderate sore throat
6	mittlere Rückenschmerzen	moderate back pain
7	mittlere Kopfschmerzen	moderate head ache
8	schwere Halsschmerzen	severe sore throat
9	schwere Rückenschmerzen	severe back pain
10	schwere Kopfschmerzen	severe head ache

Table SI5.

Side Effects

ID	German	English
1	Blähungen	Flatulence
2	Juckreiz	Itching
3	Durchfall	Diarrea
4	Fieber	Fever
5	Schwindel	Dizziness
6	Schlaflosigkeit	Insomnia
7	Sprachstörungen	Speech Disorder
8	Gedächtnisverlust	Memory Loss
9	Halluzinationen	Hallucination
10	Depression	Depression

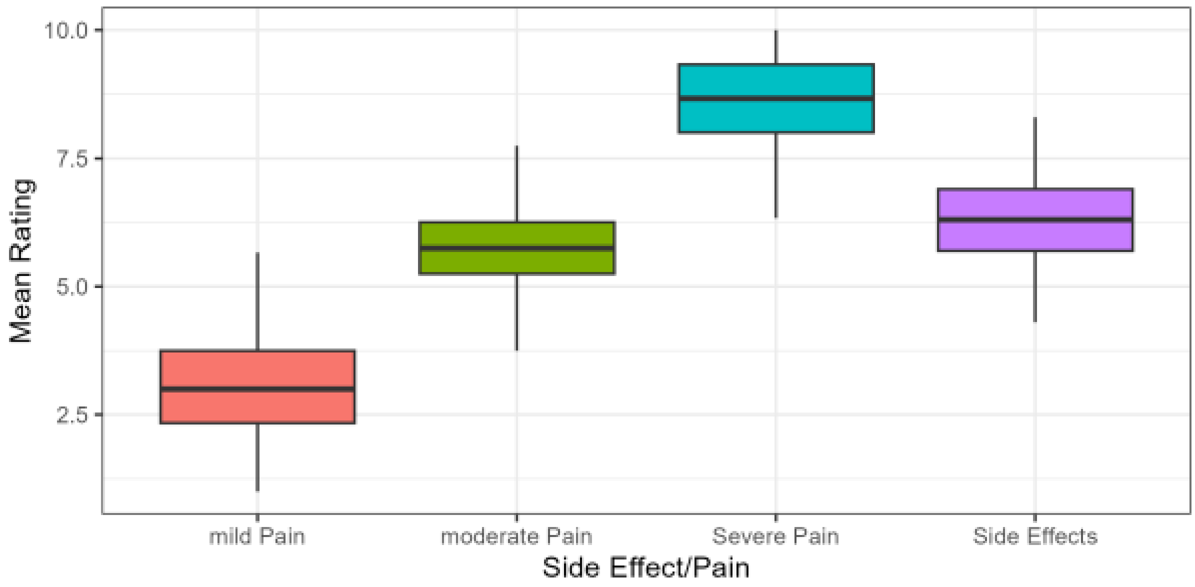

Figure SI4. Boxplots of median ratings of pains classified in mild, moderate and severe pain as well as mean side effect ratings, taken from the affect rating task over all participants.

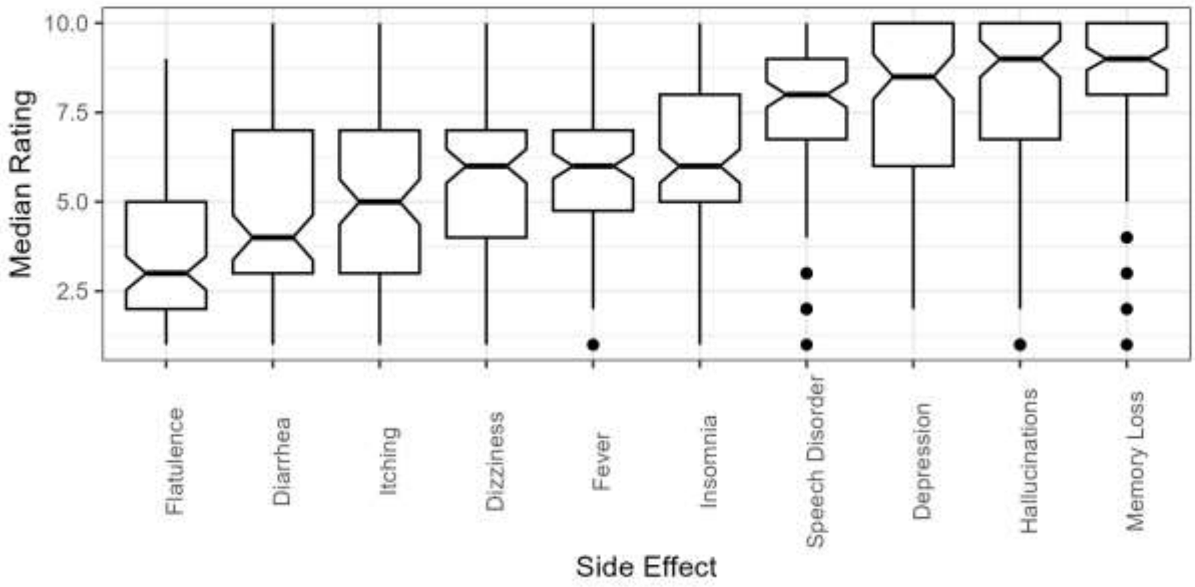

Figure SI5. Boxplots of median ratings of all types of side effects taken from the affect rating task over all participants.

5 Problem Set

Table SI6.

The following table displays the problem set used in the study.

ID	pain ID	P(A)	P(B)	A SE ID	P(A SE)	B SE ID	P(B SE)
1	2	0.880	0.733	4	0.114	2	0.178
2	8	0.680	0.614	1	0.251	8	0.051
3	9	0.905	0.709	8	0.074	1	0.205
4	3	0.545	0.694	9	0.067	10	0.026
5	9	0.921	0.730	1	0.226	5	0.089
6	1	0.644	0.914	7	0.020	3	0.140
7	2	0.688	0.706	4	0.122	10	0.024
8	9	0.635	0.611	5	0.157	6	0.092
9	2	0.511	0.778	5	0.049	8	0.054
10	2	0.675	0.775	1	0.105	4	0.176
11	8	0.836	0.703	10	0.014	6	0.185
12	1	0.922	0.563	9	0.062	6	0.056
13	7	0.584	0.658	9	0.015	8	0.027
14	5	0.804	0.889	5	0.064	3	0.163
15	9	0.642	0.902	1	0.213	8	0.042
16	7	0.828	0.810	5	0.033	2	0.104
17	8	0.645	0.800	2	0.215	1	0.188
18	5	0.517	0.835	7	0.085	9	0.072
19	8	0.900	0.786	10	0.012	3	0.181
20	7	0.676	0.571	4	0.058	1	0.218
21	5	0.769	0.681	4	0.091	6	0.219
22	5	0.86	0.782	2	0.132	10	0.014
23	7	0.596	0.944	6	0.145	7	0.135
24	3	0.575	0.724	10	0.014	5	0.183
25	4	0.791	0.631	10	0.019	9	0.019
26	9	0.752	0.624	3	0.146	8	0.037
27	9	0.561	0.824	2	0.199	4	0.151
28	4	0.767	0.871	7	0.03	7	0.117
29	2	0.766	0.635	4	0.083	3	0.136
30	7	0.609	0.691	1	0.164	2	0.081
31	4	0.754	0.833	8	0.088	4	0.235
32	7	0.713	0.763	10	0.01	9	0.041
33	8	0.659	0.594	1	0.227	6	0.134
34	6	0.770	0.620	9	0.016	6	0.091
35	3	0.645	0.677	5	0.137	7	0.017
36	8	0.569	0.795	4	0.160	10	0.071
37	8	0.566	0.766	7	0.060	4	0.192
38	3	0.860	0.659	8	0.104	3	0.138
39	9	0.571	0.514	1	0.261	10	0.014
40	8	0.711	0.536	7	0.145	1	0.139

ID	pain ID	P(A)	P(B)	A SE ID	P(A SE)	B SE ID	P(B SE)
41	4	0.845	0.869	1	0.162	6	0.068
42	6	0.556	0.715	10	0.029	7	0.105
43	9	0.606	0.577	7	0.036	3	0.105
44	7	0.703	0.756	2	0.162	5	0.103
45	2	0.693	0.708	7	0.031	2	0.180
46	8	0.794	0.837	5	0.200	10	0.018
47	9	0.741	0.571	10	0.020	1	0.198
48	6	0.733	0.799	3	0.167	9	0.038
49	1	0.812	0.817	6	0.037	2	0.199
50	2	0.626	0.571	1	0.205	8	0.027
51	3	0.742	0.688	9	0.052	5	0.112
52	3	0.595	0.669	2	0.187	6	0.065
53	9	0.668	0.846	3	0.153	6	0.082
54	1	0.716	0.676	6	0.074	1	0.176
55	4	0.832	0.903	5	0.118	1	0.193
56	8	0.793	0.637	10	0.013	5	0.089
57	3	0.604	0.514	9	0.043	7	0.071
58	9	0.703	0.597	5	0.066	2	0.158
59	7	0.596	0.736	5	0.137	9	0.075
60	2	0.761	0.716	1	0.276	10	0.056

Notes. Pain ID refers to the IDs given in Table SI3, P(A) and P(B) refer to the treatment probability of medication A and B respectively, A SE ID and B SE ID refer to the side effect of medication A and B with respect to table SI4, P(A SE) and P(B SE) refer to the probabilities of side effects for medication A and B respectively.

References

Gerlach, A. L., Andor, T., & Patzelt, J. (2008). Die Bedeutung von Unsicherheitsintoleranz für die Generalisierte Angststörung Modellüberlegungen und Entwicklung einer deutschen Version der Unsicherheitsintoleranz-Skala. *Zeitschrift Für Klinische Psychologie Und Psychotherapie*, *37*(3), 190–199. https://doi.org/10.1026/1616-3443.37.3.190

Gonzalez, R., & Wu, G. (1999). On the shape of the probability weighting function. *Cognitive Psychology*, *38*(1), 129–166. https://doi.org/10.1006/cogp.1998.0710

Stan Development Team. (2024). *Stan Modeling Language Users Guide and Reference Manual, Version 2.35*. https://mc-stan.org.

Stott, H. P. (2006). Cumulative prospect theory's functional menagerie. *Journal of Risk and Uncertainty*, *32*(2), 101–130. https://doi.org/10.1007/s11166-006-8289-6

Tversky, A., & Kahneman, D. (1992). Advances in prospect theory: Cumulative representation of uncertainty. *Journal of Risk and Uncertainty*, *5*(4), 297–323. https://doi.org/10.1007/BF00122574

van Doorn, J., Ly, A., Marsman, M., & Wagenmakers, E.‐J. (2020). Bayesian rank-based hypothesis testing for the rank sum test, the signed rank test, and Spearman's ρ. *Journal of Applied Statistics*, *47*(16), 2984–3006. https://doi.org/10.1080/02664763.2019.1709053
